# Changes in the Vaginal Microbiome and Associated Toxicities Following Radiation Therapy for Gynecologic Cancers

**DOI:** 10.3389/fcimb.2021.680038

**Published:** 2021-10-27

**Authors:** Despina Tsementzi, Rebecca Meador, Tony Eng, Pretesh Patel, Joseph Shelton, Jessica Arluck, Isabelle Scott, Mary Dolan, Namita Khanna, Konstantinos T. Konstantinidis, Deborah Watkins Bruner

**Affiliations:** ^1^ Nell Hodgson Woodruff School of Nursing, Emory University, Atlanta, GA, United States; ^2^ Radiation Oncology, Winship Cancer Institute, Emory University, Atlanta, GA, United States; ^3^ Department of Obstetrics and Gynecology, Emory University, Atlanta, GA, United States; ^4^ Grady Memorial Hospital, Atlanta, GA, United States; ^5^ School of Biological Sciences, Georgia Institute of Technology, Atlanta, GA, United States; ^6^ School of Civil & Environmental Engineering, Georgia Institute of Technology, Atlanta, GA, United States

**Keywords:** vaginal microbiome, postmenopausal women, gynecologic cancer, radiation toxicities, longitudinal dynamics, vaginal microbial community

## Abstract

Postmenopausal women often suffer from vaginal symptoms associated with atrophic vaginitis. Additionally, gynecologic cancer survivors may live for decades with additional, clinically significant, persistent vaginal toxicities caused by cancer therapies, including pain, dyspareunia, and sexual dysfunction. The vaginal microbiome (VM) has been previously linked with vaginal symptoms related to menopause (*i.e.* dryness). Our previous work showed that gynecologic cancer patients exhibit distinct VM profiles from healthy women, with low abundance of lactobacilli and prevalence of multiple opportunistic pathogenic bacteria. Here we explore the association between the dynamics and structure of the vaginal microbiome with the manifestation and persistence of vaginal symptoms, during one year after completion of cancer therapies, while controlling for clinical and sociodemographic factors. We compared cross-sectionally the vaginal microbiome in 134 women, 64 gynecologic patients treated with radiotherapy and 68 healthy controls, and we longitudinally followed a subset of 52 women quarterly (4 times in a year: pre-radiation therapy, 2, 6 and 12 months post-therapy). Differences among the VM profiles of cancer and healthy women were more pronounced with the progression of time. Cancer patients had higher diversity VMs and a variety of vaginal community types (CTs) that are not dominated by *Lactobacilli*, with extensive VM variation between individuals. Additionally, cancer patients exhibit highly unstable VMs (based on Bray-Curtis distances) compared to healthy controls. Vaginal symptoms prevalent in cancer patients included vaginal pain (40%), hemorrhage (35%), vaginismus (28%) and inflammation (20%), while symptoms such as dryness (45%), lack of lubrication (33%) and dyspareunia (32%) were equally or more prominent in healthy women at baseline. However, 24% of cancer patients experienced persistent symptoms at all time points, as opposed to 12% of healthy women. Symptom persistence was strongly inversely correlated with VM stability; for example, patients with persistent dryness or abnormally high pH have the most unstable microbiomes. Associations were identified between vaginal symptoms and individual bacterial taxa, including: *Prevotella* with vaginal dryness, *Delftia* with pain following vaginal intercourse, and *Gemillaceaea* with low levels of lubrication during intercourse. Taken together our results indicate that gynecologic cancer therapy is associated with reduced vaginal microbiome stability and vaginal symptom persistence.

## Introduction

Vaginal symptoms that are highly pervasive (reported by 40%-60%) in postmenopausal women (Huang et al., 2010; Palacios et al., 2018) include dryness, burning, itching, vaginal discomfort, pain and burning when urinating, dyspareunia, recurrent urinary tract infections and spotting during intercourse. Several of those symptoms are further exacerbated in gynecologic cancer patients (which are typically already menopausal), as cancer therapies pose a significant disturbance in the vaginal environment. Two-thirds of women who receive effective cancer treatments report significant treatment-related toxicities including vaginitis, fibrosis, spotting/bleeding on exam or sex, dyspareunia, and sexual dysfunction (Dandapani et al., 2015; Campbell et al., 2019). Additionally, recurrent gynecologic cancer affects around 10-74% of patients within the first 2 years following treatment (Peiretti et al., 2012; Byun et al., 2018), depending on cancer stage and various risk factors, which introduces additional rounds of therapy and further complicates treatment-related toxicities (Campbell et al., 2019). Surgery (abdominal hysterectomy +/salpingo-oophorectomy) and chemotherapy are associated with pelvic toxicities and a variety of patient-reported symptoms, most of which are acute effects diminishing over time. In contrast, radiation therapy, the most common treatment modality for cervical and endometrial cancers, continues to exert biological effects years after treatment (Dandapani et al., 2015; Smits et al., 2017). Radiation therapy is associated with vaginal toxicities including loss of lubrication, dyspareunia, vaginal itching, discharge, and cystitis, all of which may lead to increased vaginal discomfort, infection, diminished sexual activity and decreased quality of life (Leroy et al., 2012; Klopp et al., 2014; Karabuga et al., 2015).

Recent studies suggest a key role of the vaginal microbiome (VM) in symptom manifestation. Typically in asymptomatic women the VM is dominated by *Lactobacilli* and acts as the first line of defense against vaginal infections such as bacterial vaginosis, sexually transmitted infections (STIs), and urinary tract infections (Stapleton, 2016; Chehoud et al., 2017; Fuochi et al., 2017; Campisciano et al., 2018; Łaniewski et al., 2020). The VM in asymptomatic healthy women of reproductive age has two major characteristics: (a) low bacterial diversity with typical dominance of a few lactobacilli species and (b) high temporal stability (Nunn and Forney, 2016; Greenbaum et al., 2019; Berman et al., 2020). In contrast to other human organs like the gut, where high diversity is linked to healthy states, vaginal microbial communities of low diversity are associated with stability and resilience. Stability of microbial communities is a key factor in ecosystem functioning and can be quantified within two dimensions: resilience, which is the ability of community to return to the baseline state after a perturbation causes shifts (i.e. therapy interventions) and resistance, which is the capacity of the community to remain stable upon perturbations without significant shifts (Hickey et al., 2012).

In the vaginal environment, high diversity is typically associated with instability and dysbiotic states. It is believed that estrogen is the major factor contributing to this phenomenon (Romero et al., 2014; Greenbaum et al., 2019): high estrogen levels promote glycogen production from the epithelial cells, which in turn grants advantage to lactobacilli to dominate the microbial community (Ayre, 1951; Patton et al., 2000; Mirmonsef et al., 2016). When estrogen is depleted, as it is postmenopause, the equilibrium state of the stable and low diversity community is disrupted and eventually can lead to a dysbiotic state. However, this explanation fails to account for two observations. Firstly, albeit less frequent, many postmenopausal women have been observed to maintain low diversity *Lactobacillus* dominant communities (Hummelen et al., 2011; Brotman et al., 2014). Second, some groups of both reproductive age and postmenopausal women have been observed to lack lactobacilli dominant communities, while being asymptomatic, indicating that a different equilibrium state can exist, and may vary by race/ethnicity (Wells et al., 2020). In addition, gaps in the literature related to the VM and associations with symptoms in postmenopausal women remain. For example, one review of postmenopausal vaginal communities determined they were poor in *Lactobacillus* but rich in anaerobic taxa (e.g., *Bacteroides*, *Mobiluncus*. *G. vaginalis)* (Greenbaum et al., 2019). Conversely, others have observed that in more than 50% of postmenopausal women the dominating bacteria is *Lactobacillus*, irrespective of symptoms such as vaginal dryness or vulvovaginal atrophy (Hummelen et al., 2011; Brotman et al., 2014). Nevertheless, in both later studies, a low abundance of *Lactobacillus* was still associated with vaginal symptoms.

Additionally the VM is believed to be involved in gynecologic cancers and cancer treatments. Recent studies are investigating whether or not cancer changes the microbiome or whether changes in the microbiome promote cancer (Chase et al., 2015; Contreras et al., 2016; Champer et al., 2018). In any case, the microbiome can act as an inducer of DNA damage, regulating cell growth and death and modulating host immune responses (Wang et al., 2017). In addition, and as our own work suggests (Tsementzi et al., 2020), cancer therapies may be associated with significant changes in the VM (Bhatt et al., 2017; Muls et al., 2017). Based on such evidence, it is hypothesized that the VM might be a critical contributor to the manifestation of pathologic states in the vaginal environment, particularly in women with gynecologic cancer who experience cancer treatment interventions.

In our previous preliminary work, we examined the VM of gynecologic cancer patients from baseline pre -radiation therapy to 2-4 months post-radiation therapy as compared to healthy controls and found that significant differences exist between the two groups (Tsementzi et al., 2020). Cancer patients have higher diversity microbiomes with multiple anaerobic, and potentially pathogenic, species compared to healthy women of similar age, self-reported race and body mass index (BMI). Here we expand on this work to investigate the temporal dynamics of the VM in a time series up to 1 year post-RT, and their associations with vaginal toxicities and symptoms. We hypothesized that that differences in species composition may correlate with how vaginal communities respond to disturbances, and the magnitude of that response (stability) might be associated with manifestations of symptoms, indicating a link between stability and ecosystem functioning. We examine the shifts of VM communities during and up to a year after the completion of cancer treatments, and we compare those changes with those identified in healthy women without apparent interventions. Finally, we aim to identify VM characteristics that may be associated with vaginal symptoms, while accounting for sociodemographic, clinical and behavioral data that potentially affect the VM.

## Materials and Methods

### Study Design

The study was approved by Emory University’s Institutional Review Board, and informed consent was obtained from each participant. For the cancer cohort, postmenopausal women (naturally or due to surgical menopause, i.e., radical hysterectomy/removal of ovaries) with Stage IB-IIIC endometrial or cervical cancer that had been scheduled to receive radiation therapy with curative intent (external beam radiation therapy and/or brachytherapy) were invited to participate in the study. Healthy postmenopausal women without gynecologic cancers were invited to participate during their routine annual gynecologic visit. The healthy cohort was selected to match the cancer cohort in terms of the age, BMI and self-reported race/ethnicity. Exclusion criteria included history of metastatic or other primary cancer and comorbidities that may cause severe vaginal toxicities (e.g., HIV, cystic fibrosis, Type I diabetes or poorly controlled Type II diabetes, autoimmune disease, current STIs, HSV, hepatitis C, and use of interferon or immunosuppressive therapies) and the use of antibiotics or corticosteroids within 4 weeks prior to baseline assessment. Study participants were recruited within a period of 3 years (6/2016-3/2019) and all sample collection was completed by 3/2020.

A total of 134 women were recruited in the study and had samples taken at baseline (T0): 64 gynecologic cancer patients and 68 healthy participants. Among them, 52 participants (25 gynecologic cancer patients and 27 healthy women) agreed to follow up assessments and had their samples taken at an additional 3 time points past the baseline, in order to quantify long term dynamics in the vaginal microbiome. For cancer patients, the first sample was collected after cancer diagnosis and at least 4 weeks after surgery (for those patients for which surgery was prescribed) and prior to the start of radiation therapy (T0). Subsequently samples were taken 2 to 3.5 months past baseline, at which time all radiation therapy sessions had been completed (T1). The post-radiation samples were typically collected 2.5-3 weeks after the last radiotherapy session. Additionally, samples were collected at 6 months (T2) and 1 year (T3) past the baseline. Follow up samplings and evaluations were performed at the clinics for cancer patients during regularly scheduled follow-ups with their radiation oncologist. Healthy subjects were also sampled using the same time frame to account for maturation effects or other changes of the vaginal microbiome during the course of the year. For healthy participants, since they do not typically return to the clinic after their annual evaluation, follow up samplings were performed using self-collection kits that were sent to the participants’ homes. Detailed written and pictorial instructions on how to use the vaginal swabs, how to complete patient reported questionnaires, and how to store and return samples in the pre-paid shipping boxes were included in the self-collection kits. Baseline VM samples and have been previously reported (Tsementzi et al., 2020).

### Assessment and Evaluation of Clinical, Demographic, and Behavioral Factors

Clinical information (cancer type and stage, treatment type, BMI, age, vaginal pH), sociodemographic factors (self-identified race), and lifestyle habits (smoking, alcohol consumption) were recorded from the recruited volunteers and/or from their medical history during the baseline sampling. Vaginal pH was measured for all participants at each sampling, using sterile polyester swabs, which were rolled onto pH strips (Merck, Darmstadt, Germany) and scored from 4.0 - 7.7 according to the manufacturer instructions. Ph below 5 was categorized as “normal” and above 5 as “high”.

Typical vaginal practices and other behaviors that might affect the vaginal microbiome were evaluated at baseline and at subsequent sampling points by asking all participants for their use of any of the following during the past 4 weeks: antibiotics, corticosteroids, topical estrogen and hormonal replacement therapy, vaginal douching, lubricants/moisturizers, vaginal probiotics and sexual intercourse.

Vaginal toxicities commonly caused by radiation therapy were evaluated using the clinician-reported Common Terminology Criteria for Adverse Event (CTCAE) Reporting System criteria (v5.0) items for vaginal toxicities. The NCI CTCAE is a grading (severity) scale for adverse events (AE), with each item scored on a scale from 0 (no discomfort) to 3 (severe discomfort). *Six vaginal-related toxicities* were rated by clinicians: *dyspareunia, vaginal pain, vaginal dryness, hemorrhage, inflammation, and vaginismus*. For the vast majority of participants, the clinician-rated CTCAE scores were only rated from 0 (no discomfort) to 1 (mild) and 2 (moderate discomfort), thus the scoring system was transformed from ordinary to categorical variables (presence [score of 1-2] or absence [score of 0]) to achieve higher statistical resolution due to the small sample size available. CTCAE based toxicities were reported for all subjects at baseline, and only for cancer patients during their follow-ups at the clinics (healthy subjects did not require additional physician visits within one year and thus performed self-collections at follow-ups). The term toxicities is used throughout the manuscript to describe the aforementioned symptoms which have been scored by clinicians.

Vaginal symptoms commonly reported by postmenopausal women were evaluated at baseline and follow up assessments for all participants, using patient-reported questionnaires. Using a format modeled on the severity items of the Patient Reported Outcomes version of the CTCAE, the PRO-CTCAE (Dueck et al., 2015), each participant was asked if they experienced any of the following *four vaginal symptoms*, at the time of sampling, rated on a scale of 0 (none) to 4 (very severe): *dryness, potential yeast infection (itching), discharge or bleeding*. Finally, two additional symptom were quantified for all sexually active participants, which were asked to complete the Female Sexual Function Index (FSFI) questionnaire for evaluating sexual dysfunction (Rosen et al., 2000; Meston, 2003). The FSFI questionnaire contains 19 validated items that comprise six domains including sexual desire, arousal, lubrication, orgasm, satisfaction, and pain. Each domain is rated on a scale of 0 (no dysfunction) to 6 (severe dysfunction), and the composite FSFI score is an additive of all domains ranging from 0 to 36. A cutoff of 26 has been previously validated to differentiate between subjects with and without sexual dysfunction. In this study, the items pertaining to *lack of lubrication* and *vaginal pain during intercourse* were used as proxies of vaginal health and treated as ordinal variables in subsequent analysis.

For the longitudinal analysis of the subset of women for which time series samples were taken (n=52), frequency of symptoms was calculated based on the individual patient reports from the four time points. Patients’ symptom persistence was scored for each symptom as follows: 0 (never experienced symptom), once out of four times (occasional), two or three times out of the four (frequent), and at all-time points (constantly). Additionally, behavioral data (use of corticosteroids, antibiotics, topical estrogen or HRT, vaginal lubricants or moisturizers, douching and sexual activity) were treated in a similar manner, and participants were categorized on the described four categories based on the frequency of the reported behaviors.

### Vaginal Microbiome Sampling and Processing

Vaginal swab samples were collected from mid-vagina as previously described (Tsementzi et al., 2020). Once collected, all swabs were stored in the Qiagen DNeasy PowerSoil Kit buffer-containing tubes and frozen upright until they could be transported to the laboratory, where they were stored at −80 °C. Additionally, eight sampling blanks were collected at the clinics and four extraction blanks were also included during the processing steps. DNA was extracted with the DNeasy PowerSoil Kit (Qiagen) following the manufacturer’s recommendations, with minor modifications to improve DNA yield and quality: mechanical lysis by bead beating was only limited to three 5 sec vortex pulses during a 10 min 75°C incubation of the swabs with the C1 lysis buffer and with the addition of proteinase K at 50ug/ml final concentration. DNA was quantified with a qubit fluorometer, and further concentrated when needed with ethanol precipitations to achieve 5ng/ul concentrations. The V4 region of the 16S rRNA gene was amplified with the F515 and R806 primers using the previously described 2 step PCR and dual index protocol for Nextera XT (Kozich et al., 2013) and sequenced using the MiSeQ Reagent Kit v2 (Illumina). All samples were sequenced in the same run, and blanks were included in the sequencing to represent (a) sampling blanks, collected at the clinics, (b) DNA extraction blanks, one for each batch of DNA extraction and (c) PCR amplification blanks.

### 16S rRNA Gene Amplicon Sequencing and Processing

The 16S rRNA gene sequences were processed as previously described to exclude adaptor and primers sequences using cutadapt (Tsementzi et al., 2020). Trimming of low quality and chimeric sequences was done with the dada2 wrapper pipeline implemented in QIIME v2 (Bolyen et al., 2019), trimming sequences where the median quality dropped below q20. After the denoising step, reads were clustered into ASVs (Amplicon sequence Variants) with dada2. Sample coverage was calculated using the Turing Good and Chao estimator (R package vegan) (Oksanen et al., 2013). Taxonomy was assigned with the RDP classifier, trained with the Silva database (silva-132) trained for the V4 region using the classify-sklearn option in QIIME2 with a 0.8 confidence cutoff. The OTU table constructed to represent bacterial genera was normalized for sequencing depth using the cumulative sum scaling transformation (metagenomeSeq package) (Paulson et al., 2013).

### Diversity Estimates

α-diversity was estimated using four complimentary metrics implemented in the R package vegan: Chao-1 index to estimate OTU richness (number of total OTUs present in the sample), Pielou index to estimate OTU evenness (similarity of abundances across OTUs), Shannon index (evenness and richness composite), and Faith’s (PD) index to account for phylogenetic diversity. α-diversity values distribution was tested for normality with the Kolmogorov-Smirnov, and values were compared between gynecologic cancer and healthy subjects using the Kruskal-Wallis test (independent samples) and between time points using the Wilcoxon signed-rank test (dependent samples). β-diversity distances were estimated using Bray-Curtis dissimilarities (abundance weighted distance) using the R package vegan.

### Definition of Vaginal Community Types

Vaginal community types were identified as previously described (Ravel et al., 2011). Similarities in the composition of the vaginal bacterial communities were assessed by hierarchical clustering of the OTU table, which was first filtered to maintain genera with at least 0.1% abundance in at least one sample. Ward’s linkage hierarchical clustering was computed on the β-diversity distances using the hclust r function. The resulting dendrogram reflects the degree of dissimilarity among samples in terms of relative microbial species abundances. Additionally, Spearman’s correlation coefficient profile between communities were calculated, and clustering was done with the use of complete linkage in which the maximum distance between two clusters is computed as previously done (Brotman et al., 2014). Clusters were extracted using the cuttree function in R. Associations between metadata and vaginal community types were performed in R using ANOVA for continuous variables and Fisher’s exact test for categorical variables with Bonferroni corrections for multiple testing.

### Vaginal Microbial Community Stability Metrics

For the 52 participants for whom longitudinal data were available, we evaluated shifts in the microbial community composition using 3 metrics of microbiome stability. The overall stability for a vaginal microbiome community was estimated using the average of the Bray-Curtis (ABC) dissimilarity index between all samples taken from the same subject (four time points collected). Maximum Bray-Curtis distances were also evaluated and resulted in similar patterns as the average distances. Bray-Curtis distances between T0 (baseline) and the final assessment after one year (T3) were calculated as a proxy of community resilience (long-term effects in the composition). Finally, shifts between baseline and T1 (completion of therapy for cancer population) were estimated using Bray-Curtis distances and used as a proxy for community *resistance* (short-term changes after a disturbance, which in this case was assumed to be the radiation treatment for the cancer cohort).

We evaluated the relationships of the VM stability with the available metadata, as well as the prevalence and persistence of the clinician-reported vaginal toxicities and patient-reported vaginal symptoms. For this analysis, we examined three types of variables (a) constant throughout the year (i.e. age, BMI, race, cohort, cancer type, etc.), (b) one-time events at baseline (i.e. reported symptoms or behaviors at baseline), and (c) longitudinal data obtained during four sampling points (i.e., symptom persistence, persistence in behaviors such as topical estrogen use and antibiotics).

For each of the three metrics of stability, we categorized the vaginal microbiomes into three groups (high, intermediate and low stability ranging from 0-0.5, 0.5-0.75, 0.75-1 in any of the Bray-Curtis stability metrics accordingly). Associations between the stability levels and metadata were initially tested with unadjusted Fisher’s exact tests and Kruskal-Wallis tests for comparing the values between cancer and healthy groups. Subsequently, we used a forward selection of the linear model in order to identify the best explanatory variables for the distribution of each of the stability metrics. For this analysis, we only used metadata with no missing values. We used a generalized linear model as implement in the glm function of R. The order of the variables was selected by the Akaike information criterion with forward model selection, as implemented in the “step” function in R. Finally, an ANOVA was run on the glm model using the anova.glm function in R.

### Reference Vaginal Microbiome Datasets From Reproductive Age Women

Vaginal microbiome longitudinal data from reproductive age women were used as a reference to assess differences in the magnitude of temporal shifts in the VM in this sample of postmenopausal women. We were not able to identify longitudinal datasets that extend one year, thus we used two previously published datasets that sampled vaginal microbiomes for 3 and 4 months. In both studies the VM was sampled and sequenced with similar protocols to the ones used in the current study. The first study longitudinally evaluated the effect of menses on the composition of the vaginal microbiome, by sampling eight healthy reproductive age women 15 times during a period of 3 months (Hickey et al., 2013). The second study evaluated the temporal microbiome dynamics of 32 women and obtained 32 samples from each individual during a period of four months (Gajer et al., 2012). The raw sequence data were downloaded from the SRA archive and processed using the same pipeline as the postmenopausal vaginal microbiome data obtained here, in order to construct taxonomic distributions and evaluate metrics of stability for the vaginal microbial communities.

### Statistical Analysis

Distance matrices based on the Bray-Curtis dissimilarity index were used to conduct permutational univariate and multivariate nonparametric analysis of dissimilarities (ADONIS2) using the R package vegan and P-values were adjusted for multiple testing using the Bonferroni correction (Oksanen et al., 2013). This analysis was repeated using Jaccard, and Mahallanobis distances, and all yielded consistent results unless otherwise notyed. Frequency of vaginal symptoms and personal practices were tested for associations with either (a) the vaginal microbiome structure at baseline, using either β- diversity distances or vaginal community state types and (b) with the stability metrics of the vaginal microbiome. Student’s t-test was used to assess the statistical significance of differences in baseline characteristics and α -diversity metrics for which the data were normally distributed. Kruskal-Wallis tests were used for non-normal distributed metadata.

## Results

### Study Population Characteristics

A total of 65 gynecologic cancer patients and 69 healthy postmenopausal women were recruited in the study (**Table 1**). All patients had vaginal microbiome samples taken at baseline, as previously described (Tsementzi et al., 2020). Gynecologic cancer patients and control groups had no significant differences in age, BMI, or racial distributions (**Table 1**). At baseline, 57% of cancer patients had already completed surgery (abdominal hysterectomy +/salpingo-oophorectomy) at least 4 weeks prior to the first sampling. Additionally, 33% of cancer patients received chemotherapy, whether prior or concomitant with radiation therapy. All treatment modules were completed by the second time point (T1). The most common chemotherapy regimens were 6 cycles of Carbo/Taxol, followed by EBRT, or 5 weeks of concurrent chemoRT with Cisplatin. Healthy controls were recruited during their annual gynecologic exams, and they had no major interventions pertaining to vaginal issues recorded in their medical history.

**Table 1:** Clinical and demographic information of cancer patients and healthy controls.

	Cancer T: n=65	Healthy T: n=69	Total T: n=134	P-value (cancer/healthy)
	S: n= 25	S: n=27	S: n=52	
**Age years, mean [SD]**	T: 56.1 [13.4] S: 57.5 [9.9]	T: 59.3 [7.8] S: 60.1 [9.9]	T: 57.9 [11] S: 58.5 [10.0]	T: 0.08 (Unpaired T-test) S: 0.09 (Unpaired T-test)
**BMI, mean [SD]**	T: 31.4 [7.6] S: 29.1 [7.7]	T: 28.7 [7.9] S: 29.4 [7.4]	T: 30.1 [7.8] S: 29.1 [7.6]	T: 0.02 (Kruskal-Wallis) S: 0.02 (Kruskal-Wallis)
**Self-reported race, n (%)**				
Caucasian	T: 29 (44.6%) T: 14 (56.0%)	T: 31 (44.9%) T: 17 (63.0%)	T: 60 (44.8%) T: 31 (59.6%)	T: 0.51 (Fisher-exact) S: 0.57 (Fisher-exact)
African American	T: 34 (52.3%) T: 10 (40.0%)	T: 33 (47.8%) T: 9 (33.3%)	T: 67 (50.0%) T: 19 (36.5%)	
Asian	T: 2 (3.1%) T: 1 (4.0%)	T: 5 (7.2%) T: 1 (3.7%)	T: 7 (5.2%) T: 2 (3.8%)	
				
**Diagnosis, n (%)**				
Endometrial	T: 36 (55.4%) S: 14 (56.0%)			
Cervical	T: 29 (44.6%) S: 11 (44.0%)			
				
**Type of Treatment at T0**	**T: n=65 | S: n=25**			
Surgery	T: 30 (46%) | T: 13 (52%)			
Surgery + Chemotherapy	T: 7 (11%) | T: 3 (12%)			
None	T: 18 (27%) | T: 9 (36%)			
**Type of Treatment at T1**	**S: n=25**			
Radiation	11 (44%)			
Radiation + Chemotherapy	14 (66%)			
None	0 (0%)			

Table depicts all subjects included in the study. After quality assurance, a total of six datasets were excluded due to low quality, thus 124 subjects had VM at T0. For a subset of those subjects (n=52), longitudinal data were obtained (depicted in grey color). After excluding low quality datasets, 42 subjects (22 cancer and 20 healthy) had longitudinal VM data at four time points. Summary statistics are given for the total population recruited in the study and had baseline data analyzed (T) as well as for the subset of patients that had longitudinal data available (S).

### Vaginal Toxicities and Symptoms in Gynecologic Cancer and Healthy Postmenopausal Women

**Figure 1:**
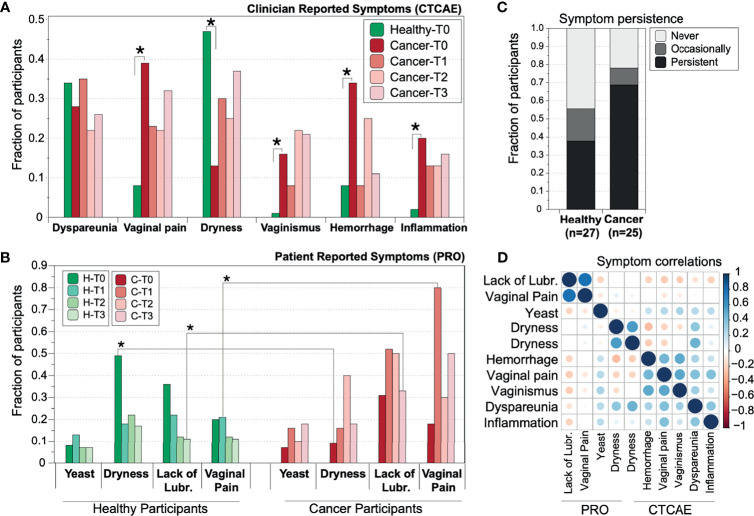
Prevalence of reported vaginal symptoms among gynecologic cancer and healthy postmenopausal women at the different time points of the study. **(A)** Prevalence of clinician-reported toxicities **(B)** Prevalence of patient-reported outcomes (PROs)/symptoms. T0=baseline (n=134); T1 = 2.5-3mos after completion of radiation therapy (n=52), T2 = 6mos (n=52), T3 = 12mos post baseline (n=52). Star symbols indicate statistical significance (p=<0.01, z-test with correction for multiple testing) of the different proportion between gynecologic cancer and healthy cohort. **(C)** Vaginal symptoms persistence in gynecologic cancer and healthy controls. Percentage of healthy and cancer participants which reported at least one of the four patient reported symptoms: Never, Occasionally (once or twice in the year), Persistently (three or four out of four times). **(D)** Correlation of PRO symptoms. The correlations were tested after transforming all data to categorical variables (presence or absence of symptom).

Vaginal symptomatology reported by clinicians included *dyspareunia, vaginal pain, dryness, vaginismus, hemorrhage, and inflammation* (**Figure 1**). Patient reported symptoms included *dryness* and *yeast infection* (itching and/or discharge), and for participants who were sexually active (58% of healthy women and 46% of gynecologic cancer patients), *lack of lubrication during sexual intercourse* and *vaginal pain during sexual intercourse (*aka patient reported dyspareunia, FSFI item*)* were also reported.

#### Clinician-Reported Vaginal Toxicities

At baseline, clinicians reported that cancer patients had significantly higher instances of vaginal pain (p=0.002, adjusted z-test), bleeding (p=0.004), vaginismus (p=0.0001), and vaginal inflammation (p=0.009). All four clinician-reported toxicities were consistently reported at similar, albeit slightly lower levels at the subsequent time points and up to one year (**Figure 1A**) following treatment. In healthy controls, the most commonly reported symptom was vaginal dryness, which affected 45% of healthy controls. In contrast, cancer patients reported dryness at significantly lower levels (16%, p=0.008). However, persistence of dryness seemed to significantly decline for the healthy cohort after 3 (T1), 6 (T2) and 12 (T3) months from baseline, while it remained at the same or higher levels for those cancer patients who were experiencing dryness at baseline (**Figure 1B**).

#### Patient-Reported Vaginal Symptoms

Vaginal dryness, as reported by the participants, was found to be more prominent in healthy controls at baseline (**Figure 1**). However, persistence of dryness seemed to significantly decline for the healthy cohort after 3 (T1), 6 (T2) and 12 (T3) months from baseline, while it remained at the same or higher levels for those cancer patients who were experiencing dryness at baseline (**Figure 1B**). Gynecologic cancer patients self-reported more vaginal pain and lack of lubrication during sexual intercourse at subsequent time points compared to baseline. A symptom was reported as present on those subjects who scored lower than 4.2 out of six in the corresponding FSFI domain score (SI **Figure 1**). Vaginal pain was prominent in about 80% of cancer patients after the completion of radiotherapy at T1, in contrast with 20% of healthy subjects (p=0.009). Lack of lubrication was reported by ~30% of all subjects at baseline but affected 50% of cancer subjects at the end of the year, while healthy women reported similar levels throughout the year (**Figure 1B** and **SI Figure 1**).

Among cancer patients, 20% self-reported at least one symptom consistently in all their evaluations, as opposed to only 12% of healthy women (**Figure 1C**). For the subset of people for which longitudinal data were available, persistence of symptoms was indeed more prominent in the cancer cohort, while 8% of healthy women only occasionally (i.e., 1 or 2 times out of the four) reported vaginal symptoms, 12% exhibit persistent symptomatology and around 20% never reported a symptom. In contrast, around 30% of cancer subjects report frequent or consistent symptoms, and only 7% never reported a symptom during the study duration.

#### Clinician Versus Patient Reports

From the symptoms investigated, dryness was scored independently by both clinicians and patients, and while discrepancies between the two reports are expected (Deshpande et al., 2011), we observed a relatively high correlation (r=0.68). Vaginal pain (reported by both clinicians and patients) and vaginismus in the same subjects were moderately correlated with a coefficient of 0.54, while all other symptoms and toxicities were not well correlated (**Figure 1D**). A slight correlation was observed between the two reported FSFI symptoms of lack of lubrication and vaginal pain during intercourse (r=0.45). This weak association might be partially explained by the small sample size, since only a subset of women was sexually active and reported FSFI scores.

### Vaginal Microbiome Dataset Quality

On average, 63% of the 16S rRNA gene amplicon reads per dataset passed the quality trimming (stdev 12.8%), after removing low quality sequences, chimeras, and duplicates (**SI Figure 2B**). In addition, we included 15 blank samples in the same sequencing run to represent sampling, extraction, and amplification negative controls. For 8 out of the 15 blanks, an average 34% of the reads passed the quality thresholds which resulted in blank datasets with <3.2K good quality reads in total (**SI Figure 2A**). For comparison, the median number of sequences for all vaginal samples was 35.2K reads. Only 10 VM datasets yielded <3.2K reads (including all blanks) and were removed from subsequent analysis, resulting in 267 high quality datasets. Among all available datasets, we identified 4164 unique features (ASVs), which corresponded to 700 bacterial genera. Out of 700 genera, 154 were found in the blanks (**SI Figure 2B**), however most of them (113) were found in only 1 out of 8 samples, and 50% of the genera were never observed in any other sample. We subsequently compared each blank sample with the corresponding vaginal swab sample, which was collected at the same time, and confirmed that blanks have a significantly different taxonomic distribution from the vaginal swabs, which doesn’t seem to be structured (**SI Figure 2D**). On close inspection, the most common genera that were found in the blanks were previously known contaminants, including *Escherichia*, *Strenotrophomans*, *Ralstonia* and *Corynebacterium* (Salter et al., 2014; Glassing et al., 2016; Eisenhofer et al., 2019; Weyrich et al., 2019). Thus, removing all datasets with a similarly low yield as the blanks, eliminated contamination issues that might bias the taxonomic distributions.

The estimated sample coverage for the 267 high quality vaginal microbiome datasets, i.e., the probability for a species of the community to be observed in the actual sequence dataset obtained, was nearly complete, with an average of 99.97%.

### α-Diversity of Vaginal Microbiome Communities

**Figure 2:**
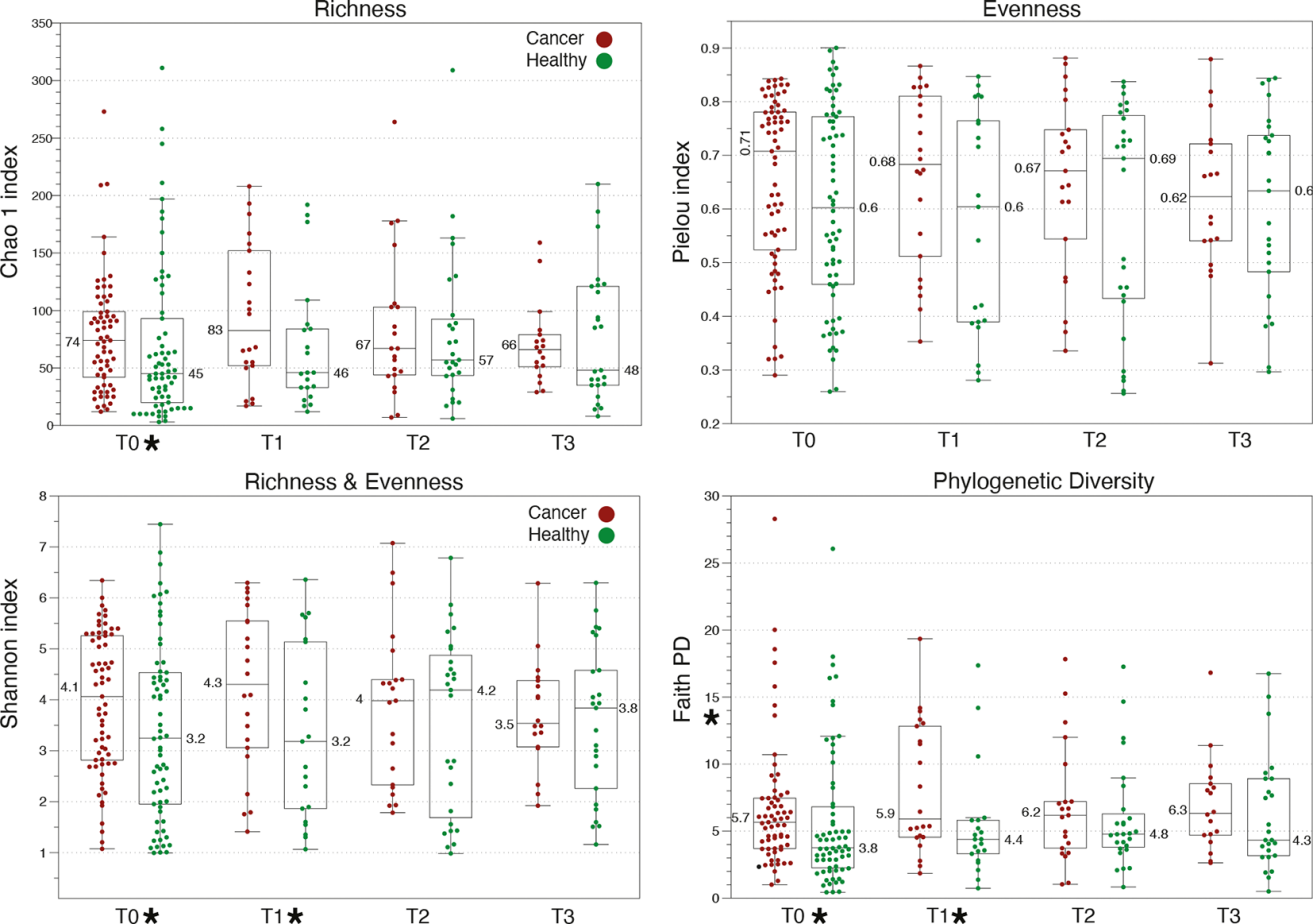
Comparison of α-diversity metrics among healthy and gynecologic cancer vaginal microbiome communities. Cancer patients exhibit higher α-diversity than healthy controls at baseline, which further increases at T1, upon completion of therapy. Star symbols indicate a p-value <0.01 in Kruskal-Wallis test between cancer and healthy.

As shown in our previous report, cancer VMs exhibit significantly higher α-diversity in comparison with healthy controls at baseline, and this difference is further exacerbated after radiation therapy. Cancer VMs had significantly higher Shannon and Phylogenetic diversity compared to healthy VMs at baseline (p=0.003 and p=0.01 rspectively) and at T1 (p=0.005 and p=0.008) (**Figure 2**). Thus, both cancer and radiation therapy have a detectable, statistically significant effect on α-diversity. However, this effect seems to be reduced in subsequent time points, in which α -diversity is reduced for cancer patients and returns to similar levels as the healthy controls (**Figure 2**). At T2 and T3, there were no statistically significant differences in the VM between cancer patients and healthy controls. There is a clear reduction trend in α-diversity with the passing of time for cancer patients in contrast to healthy individuals who maintained similar levels of α -diversity throughout the year. Consistent with our previous observations, we didn’t identify any α-diversity differences when comparing the VMs of endometrial cancer patients with the VMs of cervical cancer patients or when the comparisons were performed among Caucasian and African American women, at either baseline or subsequent sampling points.

### VM Community Structure in Cancer and Healthy Vaginal Samples

**Figure 3:**
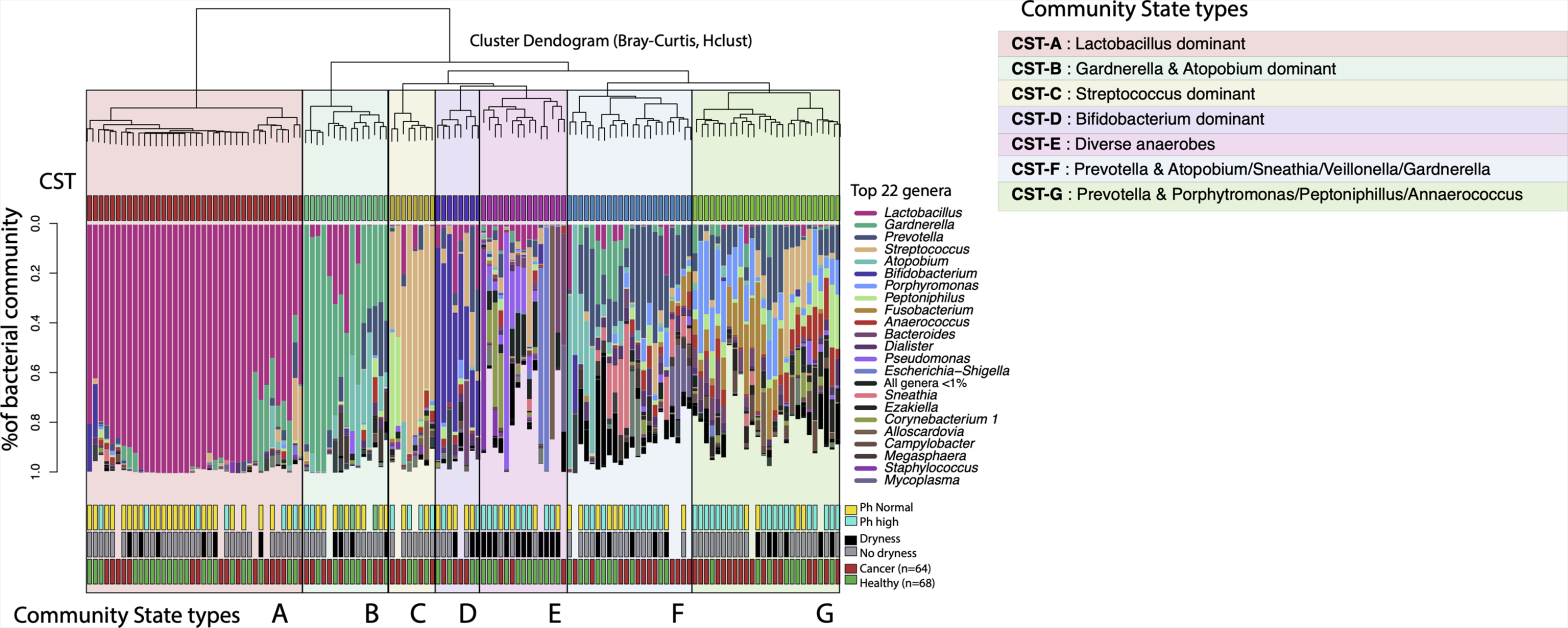
Vaginal microbiome community state types (CST-A) identified in gynecologic cancer and healthy postmenopausal women. The dendrogram represents the hierarchical clustering of the VM communities based on Bray-Curtis dissimilarity distances. The taxonomic profiles for each VM are presented in stacked barplots for each subject, where only the most abundant 22 bacterial genera are shown (for aiding visualization). Certain CSTs are highly correlated with toxicities/symptoms, for example CST-E shows high correlation with persistent vaginal dryness (Fisher’s exact test and Bonferroni correction, p=0.001).

We identified 7 distinct vaginal community clusters or types (CTs) among all postmenopausal women in this study (**Figure 3**). The most prominent type, CT-A, was dominated by *Lactobacillus* species (i.e. >95% abundance in 80% of the CT-A type microbiomes) and was identified in 28.6% of our study cohort (**Table 2**). Two community state types (CT-F and CT-G) were characterized by the prevalence of the *Prevotella* genus and were found in about 26% of the women. The two types were differentiated based on the presence and dominance of *Atopobium*, *Sneathia*, *Veillonella* and *Gardnerella* species for CT-F, and *Porhyromonas*, *Peptoniphillus, Fusobacterium* and *Annaerococcus* species for the CT-G type. About 11% of women were categorized within CT-B, a state type characterized by high abundance of *Gardnerella* and *Atopobium*, but with the absence of *Prevotella*. Types CT-C and CT-D were the rarest ones, with each being found in only about 6% of the women. The CT-C was characterized by high abundance of *Streptococcus*, dominating >40% of the total microbial community. Type CT-D was characterized by dominance of *Bifidobacterium* species, found in 25% abundance over the total vaginal community. Finally, the most diverse type, CT-E was found in 15% of women and included vaginal microbiomes with various diverse anaerobes, without any single taxon dominating; these VMs were clustered together due to high diversity and lack of resemblance with the other types. Notably, high diversity VMs with diverse anaerobes are typically linked with dysbiotic states (Nunn and Forney, 2016).

**Table 2:** Vaginal microbiome community types (CT) identified in healthy and gynecologic cancer postmenopausal women.

VM	Total subjects n=124	Healthy n=20	Cancer N=22	P-value
Community State Type	# (% over total)	# (% of CT)	# (% of CT)	Fisher’s test
A	38 (28.6%)	24 (63.2%)	14 (36.8%)	0.112
B	15 (11.3%)	7 (46.7%)	8 (53.3%)	0.362
C	8 (6%)	3 (37.5%)	5 (62.5%)	0.486
D	8 (6%)	4 (50%)	4 (50%)	1
E	15 (11.3%)	3 (20%)	12 (80%)	0.0125
F	23 (17.3%)	11 (47.8%)	12 (52.2%)	0.653
G	26 (19.5%)	9 (34.6%)	17 (65.4%)	0.0789

Among all types, the high diversity CT-E, and the *Prevotella* and *Porphyromonas* dominated CT-G type were found more commonly found among the cancer cohort (80% and 60% respectively) compared to the healthy controls (**Table 2**). All other types seemed to be equally distributed between cancer and healthy subjects. Additionally, no differences were observed in the distribution of cervicotypes among endometrial and cervical cancer, or different treatment modules.

### Effects of Clinical and Demographics Factors on the VM

In our previous publication, we concluded that the most significant predictors of the VM structure include the subject (intra-person variation), cohort (cancer vs health), abnormally high pH, and age (Tsementzi et al., 2020). In this study, we included additional behavioral data and identified that both sexual intercourse and use of topical estrogen during the 4 weeks prior to sampling were significantly associated with the differences observed on the VMs at baseline.

Using the VM community state types, we aimed to identify factors that influence the VM structure (i.e. distribution of vaginal community types). Consistent with previous results, pH and age were significantly associated with the community types (**Table 3**), while BMI and race/ethnicity had no effect. The *Lactobacillus* dominant CT-A type was typically associated with the use of topical estrogen, younger ages, and lower pH levels (**Figure 3**). On the other hand, the high diversity types CT-F, G and E were associated with sexual intercourse and abnormally high pH levels.

**Table 3:** Associations of vaginal symptoms and clinical data with identified vaginal community state types in cancer and healthy postmenopausal women.

Evaluation of associations	p-value
***Anova test***	
Age	0.017*
BMI	0.462
***Fisher’s test***	
Race	0.378
HRT use	0.047*
Smoking	0.473
Ph category (normal *vs* high)	0.0004*
Sexual intercourse in the past 4w	0.029*
**Symptoms (Yes/No)**	
Dyspareunia	0.234
Vaginal Pain	0.23
Vaginal Dryness	0.08
Vaginismus	0.68
Vaginal Inflammation	0.11
Yeast Infection	0.059
Dryness (PRO)	0.0001*
Discharge	0.21
Bleeding	0.87

*denotes P < 0.01.

### Vaginal Microbiome and Relationship With Reported Vaginal Symptoms

Using data from baseline, we evaluated associations between the eight different vaginal symptoms/toxicities reported (**Figure 1**) with identified vaginal community state. Dryness was the only statistically significant symptom identified to highly correlate with VM at the community type level. In particular, CT-A was inversely correlated with dryness (p=0.001) (**Table 3**).

We subsequently performed multivariate analysis of variance (MaAslin), using the individual identified OTUs, and sought to investigate associations with reported symptoms. We identified six key species that showed high associations with reported symptoms among all postmenopausal women and were independent of the cohort (**SI Table 1**). *Lactobacillus* sp. correlated with low pH (p=0.003) and *Prevotella intermedia* and *Fusobacteriales* species correlated with high pH (p=0.0001). Low abundance of *Lactobacillus* sp. is associated with increased severity of vaginal dryness regardless of age, cancer status, or race (n=61, p=0.003). *Prevotella intermedia* (and to a lesser extent other *Prevotella* sp.) strongly associated with vaginal dryness (clinician-reported CTCAE) (p=0.0004). *Delftia* sp. associated with high discomfort or pain following vaginal intercourse (p=0.0006), and unclassified members of the *Gemillaceaea* family correlated with low levels of lubrication during intercourse (p=0.0002) (patient-reported FSFI).

### Longitudinal Dynamics of VM Stability in Postmenopausal Women

The magnitude of shifts in the microbial community composition was quantified for all vaginal microbiomes for which longitudinal data were available. We established three metrics of stability (overall stability, resistance, and resilience) for the microbial communities by leveraging the Bray-Curtis dissimilarity distances between samples from the same subject.

**Figure 4:**
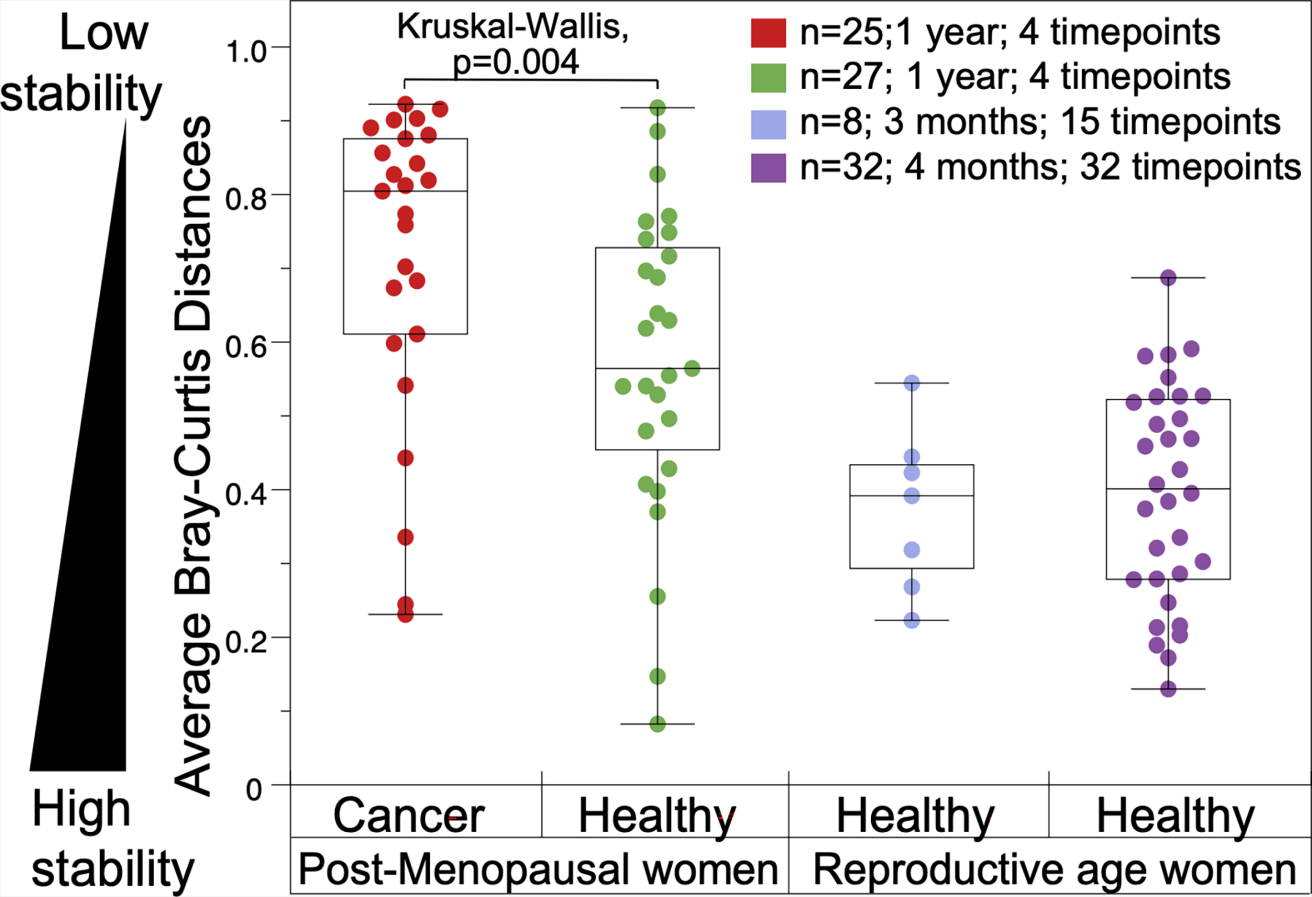
Comparison of vaginal microbiome stability in gynecologic cancer and healthy postmenopausal subjects throughout a year (on the left). (On the right), vaginal microbiomes from reproductive age women from published literature: eight healthy reproductive age women sampled over 3 months (R. J. Hickey et al., 2013); and 32 women sampled over four months (Gajer et al., 2012).

First, we estimated the overall stability of a VM as the average Bray-Curtis distance between all four time points and compared this metric among groups and a reference set from reproductive age women. Postmenopausal women in this study exhibited significantly more unstable VM communities compared to reproductive age women in the literature, and gynecologic cancer patients had the most unstable microbiomes in this comparison (**Figure 4**).

Second, we defined a metric for the resistance of the community as the distances observed between two consecutive time points (i.e. from baseline to T1). Consistent with our expectations, cancer patients show significantly lower resistance that healthy controls, as revealed by the much higher values of Bray-Curtis dissimilarities between T0 and T1 (**SI Figure 3A**). This observation could be explained by the fact that all participants in the cancer cohort were treated with radiation therapy during this time, as opposed to healthy subjects. Other potential disturbances associated to the effects of cancer could also be involved, compounded by the RT disturbance. However given that the RT is the most significant and impactful disturbance contrasting cancer and healthy subjects, it is the most parsimonious explanation that RT affects the VM stability.

Third, we sought to quantify the overall microbial community resilience, defined as the ability of the community to return to the initial stage after an intervention. We used the Bray-Curtis dissimilarity distances between T0 and T3 as a proxy for resilience. We observed similar distributions between the cancer and healthy individuals, indicating that after one year, the shifts observed in their VMs are of equal magnitude. Taken together with the low stability and resistance of the VMs from the cancer cohort, the above observations indicate that cancer patients experience significantly larger changes in their VM during the first year after cancer treatment, and their vaginal communities change to a larger extend compared to healthy subjects after one year (beta-diversity), despite the similar levels of α-diversity observed at the one year mark.

### Metadata Correlations With the Stability of the VM

In order to identify the most significant contributors on the VM stability, we first used forward selection ANOVA on complete metadata (without missing values) to construct a generalized linear model using the stability metrics as continuous dependent variables. According to the model, overall stability is strongly influenced by the cohort, and cancer patients exhibit low stability microbiomes (**Table 4**). The cohort (cancer vs. healthy) explained about 9% of the variation according to the model (p=0.001). Additionally, the frequency of sexual intercourse (recorded as having had sexual intercourse in the past 4 weeks prior to sampling, for each time point) had a statistically significant effect (p=0.059) and explained another 6.5% of the variation observed in the microbiome stability. Frequency of sexual intercourse, as well as the use of antibiotics 4 weeks prior to sampling, was also positively correlated with low stability values, and together those parameters accounted for ~10% of the variations between T0 and T1 microbiomes (resilience metric). Finally, the frequency of use of topical estrogenwas found to be positively correlated with low resistance values and could explain 8.6% of the observed variation.

**Table 4:** Forward selection ANOVA generalized linear model on factors associated with the vaginal microbiome stability metrics.

***A. stability***					
**Average BC distances**	**Type of variable**	**Df**	**Deviance**	**Pr(>Chi)**	**Variation Explained (%)**
Cohort	constant	1	2.2243	0.02929	8.77
Sexual_Interc.	longitudinal	1	2.0644	0.05949	6.55
Race	constant	3	1.9185	0.35597	5.98
Antibiotics.4W	one-time event (TO)	1	1.8722	0.31085	1.84
HRT.	longitudinal	1	1.7631	0.33485	1.71
Antibiotics.	longitudinal	1	1.7426	0.49951	0.84
BMI	constant	1	1.7273	0.56041	0.62
Age	constant	1	1.7111	0.548	0.66
Residuals	constant	38			73.03
***B. Resistance***					
**BC TO-T1**	**Type of variable**	**Df**	**Deviance**	**Pr(>Chi)**	**Variation Explained (%)**
SexuaI_Interc.	longitudinal	1	3.9387	0.08597	5.55
Cohort	constant	1	3.8318	0.24309	2.56
Race	constant	2	3.5701	0.34451	4.01
Antibiotics.4W	one-time event (T1)	1	3.178	0.06049	6.6
Age	constant	1	3.1172	0.37862	1.4
HRT.4W	one-time event (T1)	1	3.0716	0.44598	1.09
BMI	constant	1	3.062	0.72626	0.2
Residuals					21.41
***C. Resilience***					
**BC TO-T3**	**Type of variable**	**Df**	**Deviance**	**Pr(>Chi)**	**Variation Explained (%)**
HRT.	longitudinal	1	2.962	0.04758	8.61
Sexual_Interc.	longitudinal	1	2.79	0.12001	5.3
Age	constant	1	2.614	0.11572	5.43
Antibiotics.	longitudinal	1	2.5602	0.38447	1.65
Cohort	constant	1	2.5144	0.42237	1.41
Race	constant	3	2.3301	0.45901	5.68
Antibiotics.4W	one-time event (TO)	1	2.2762	0.78661	0.16
BMI	constant	1	2.2762	0.9812	0
Residuals					71.76

In order to take advantage of all available metadata (even if incomplete), we proceeded with univariate associations while adjusting for multiple testing for additional parameters. For this analysis, and for each of the three metrics, stability, resilience and resistance, we categorized participants in three groups: low, intermediate, and high. We then performed Fisher’s and Kruskal-Wallis tests to identify significant effectors on the metrics’ distributions (**SI Tables 3–5**).

Among the parameters tested, we found that the overall *stability* of the vaginal microbiome was strongly correlated with cohort (cancer *vs* healthy, p=0.045), pH level (normal or basic, p=0.044), chemotherapy intervention at T0, and with persistent dryness (p=0.0471) (**SI Table 2**). Additionally, the metric of *resilience* (differences between T0 and T3) was strongly correlated with cohort (p=0.043), chemotherapy intervention at T0, and the frequency of use of topical estrogen (0.0273) (**SI Table 3**). In other words, cancer patients, and especially those who were treated with chemotherapy before baseline (pre-radiotherapy) sampling, tend to have the most unstable microbiomes. As expected, use of topical estrogen associates with smaller microbiome changes from baseline to one year later. Finally, the short-term stability metric of *resistance* (T0-T1 comparisons) was found to highly correlate with pH levels (0.039), and persistence in the symptoms of dryness (0.0471) and yeast infection (0.032) (**SI Table 4**). This observation demonstrates that specific taxa and VM community states, which are often linked with persistent symptoms (independent of cancer), seem to show the highest variations in the short-term dynamics (i.e. T0-T1). Taken together, those results indicate that (a) cancer therapy effects might be more prominent in the long term, instead of short time scales and (b) vaginal symptoms are associated with short term instabilities in the VM.

## Discussion

### Vaginal Toxicities/Symptoms in Postmenopausal Women With Gynecologic Cancers

Postmenopausal women diagnosed with gynecologic cancers presented a different symptomatology and symptom persistence. At least 70% of women with cancer reported at least one symptom as being persistent during the time of the study compared to 38% of health women. Dryness and lack of lubrication were the only two symptoms that seemed to be reduced with time in the healthy cohort, presumably due to interventions such as use of topical estrogen or use of moisturizers and lubricants. With the exception of dryness, dyspareunia, and yeast infection, all other symptoms evaluated were significantly more prominent in women with gynecologic cancer at baseline. Among those symptoms, vaginismus, hemorrhage, and inflammation are known toxicities caused by cancer therapies and were observed at similar levels at each time point for the duration of one year after cancer therapies. Additionally, for the sexually active cancer participants, lack of lubrication and vaginal pain significantly increased over baseline then slightly improved with time, although these symptoms were still reported at much higher levels than in healthy controls. These observations are consistent with previous reports that show that radiation therapy significantly deteriorates sexual function, as measured by the FSFI scoring system (Bai et al., 2021). Additionally, while there is some gradual improvement, cancer patients are disproportionately affected by sexual dysfunction even one year after the completion of radiation, when compared to healthy women.

Among the vaginal toxicities/symptoms reported, only dryness seemed to be highly correlated with VM community types. Specifically in this study, vaginal dryness was inversely related to the lactobacilli dominant CST-A VMs, which is also consistent with the literature (Hummelen et al., 2011; Brotman et al., 2014; Shen et al., 2016). Only weak correlations were observed among the VM and toxicities/symptoms of hemorrhage, vaginismus, and pain; these weak associations could be related to the relatively small sample size used in our study.

While community types are not directly correlated with specific symptoms, we were able to identify individual OTUs that show strong associations with symptoms reported. For example, presence of *Delftia* sp. was associated with significantly higher pain scores following vaginal intercourse, and members of the *Gemillaceaea* family (unclassified species) correlated with low levels of lubrication during intercourse. Since none of those phylotypes are particularly abundant in the VMs, it is possible that the classification into community types will overlook those associations. On the other hand, *Lactobacillus* species are typically the dominant taxa when present in the VM, such as in the case of CST-A. As expected, lactobacilli abundance was found to inversely correlate with severity and presence of dryness as well as high vaginal pH. These observations indicate that vaginal symptoms might present different associations with the VM in each individual. Dryness is typically (but not always) is correlated with the lack of *Lactobacillus*, and it can be hypothesized that both dryness and lack of *Lactobacillus* are correlated with low estrogen levels.

Further, it is plausible that the presence of vaginal symptoms is not always correlated with a specific community structure, but rather with some equilibrium states that are maintained in an otherwise healthy vagina and varies among individuals, as our data suggests.

#### Vaginal Microbiome Structure in Postmenopausal Women

It has been well documented that the vaginal microbiome is drastically changing after menopause for the majority of women, driven by the reduction of estrogen and subsequent depletion of lactobacilli (Muhleisen and Herbst-Kralovetz, 2016; Nunn and Forney, 2016). This effect typically leads to higher diversity vaginal communities, in which the lack of a dominant species (lactobacilli) allows for the establishment of multiple lower abundance species. While the α-diversity of a community is only a proxy for a community’s status, unexpectedly high values in vaginal communities are quite often implicated with a lack of equilibrium, and often with dysbiotic states (Nunn and Forney, 2016; Brooks et al., 2017; Greenbaum et al., 2019). It is noteworthy that cancer patients show significantly higher α-diversity, which seems to be reduced, at least to levels observed in healthy women, within one year following treatment.

Both α-diversity and the taxonomic distributions of the vaginal communities also showed extremely high variability among women. Less than 30% of postmenopausal women had a low diversity vaginal microbiome dominated by *Lactobacillus* spp. species (**Figure 3** and **Table 2**). The majority of women presented high diversity and lactobacilli deficient communities, which were categorized into six distinct vaginal community types (**Figure 3**), including two types with high abundance of *Prevotella* (CST-F and G), and one type with dominance of *Gardnerella* and *Atopobium* species (CST-B). Finally, about 15% of women were classified in community type CST-E (**Figure 3**), in which no single genus was prevalent, but rather a collection of multiple diverse anaerobic genera were found with high evenness. Typically, such diverse communities are linked to pathologic states, and consistent with this expectation, vaginal microbiomes of CST-E type had abnormally highly pH values (**Figure 3**). Among the parameters recorded, we found younger ages and use of topical estrogen to be more prominent among the lactobacillus-dominant CST-A. This result is not surprising as it has been shown that the loss of estrogen is gradual when entering menopause, and younger women are expected to resemble more reproductive age VMs (Brotman et al., 2014; Muhleisen and Herbst-Kralovetz, 2016). Additionally, it has been previously reported that the use of topical estrogen can, in some cases, restore VM communities to lactobacilli dominance (Shen et al., 2016). It is important to note that while the use of estrogen and age can explain the prevalence of lactobacilli for some cases, many women belonging to the CST-A type are significantly older and report no use of estrogen. Thus, the abundance of lactobacilli in such cases points to other factors that have not been documented here. No other parameters tested were found to associate with the distribution of community state types, including the BMI, which could result in high levels of systemic estrogen in obese women.

All seven of the identified community types were found in both cancer and healthy subjects, however, the high diversity and typically low acidity type CST-E, as well as CST-G, dominated by *Prevotella* and *Porphyromonas*, were more prominent among the cancer cohort. While it is not expected that gynecologic cancer and/or therapies will cause a divergence of the VMs into a single type, it is still interesting that cancer patients more commonly exhibit VM types that might be considered dysbiotic, such as CST-E. In our previous work, we showed that cancer VMs differ from healthy controls in community structure, and we were able to identify at least 15 phylotypes that discriminate the two groups. The vast majority of those phylotypes are typically low abundance species, a characteristic of the CST-E, which have been previously associated with dysbiotic states in the vaginal environment (Tsementzi et al., 2020).

#### Vaginal Microbiome Stability and Associations With Clinical and Behavioral Data

It has been previously reported that the vaginal microbiomes of reproductive age women are highly stable with small temporal variations, and those variations are typically associated with the menstrual cycle (Gajer et al., 2012; Brooks et al., 2017; Greenbaum et al., 2019). Here, we report for the first time the changes in the VMs in women treated for gynecologic cancer up to one year post therapy compared to healthy postmenopausal controls. We observed that the stability of the vaginal communities is significantly reduced in cancer versus controls, as well as when compared to reproductive age women (**Figure 4**). Gynecologic cancer patients exhibit the most unstable microbiomes, as quantified by average Bray-Curtis distances over the four time points studied, in comparison to healthy controls. Using three metrics of VM stability, we demonstrated that the overall stability (average of 4 time points) is significantly higher in healthy women compared to cancer cases. Similarly, the resilience of maintaining low diversity VM communities, quantified as the changes from baseline to one year later (T0-T3), is significantly higher in healthy women compared to cancer patients. Apart from cancer, which was negatively correlated with resilience, only the use of topical estrogen was found to be strongly positively correlated with resilience of the VM, and participants that consistently used topical estrogen during all 4 time points of the study tended to have the most stable vaginal communities (higher resilience).

Among the other sociodemographic and clinical factors, including sexual intercourse, race, BMI or age, no strong associations were found with the overall stability and resilience of the VM. Sexual intercourse and use of antibiotics were found to be associated with shifts in the VM when evaluating short-term dynamics. In other words, when we compared the resistance of the VM, estimated by the changes that occur from baseline to 2-3 months later, we found that antibiotics and sexual intercourse were positively correlated with low resistance. Yet, there was no significant difference in terms of microbiome resistance between cancer and healthy cohorts, an observation consistent with our previous analysis of a smaller set of cross-sectional samples (Tsementzi et al., 2020). Taken together, these results indicate that the longitudinal variability is similar between healthy and cancer subjects, when comparing the VMs 3 months after the first cancer therapy. However, cancer patients show significantly larger shifts at later time points including 6 and 12 months after radiotherapy has been completed. Additionally, the use chemotherapy in cancer patients was also found to be associated with lower stability metrics. It is plausible that the effects of radiation are manifested on a longer time scale, rather than immediately after the intervention. Alternatively, cancer treatments, which are completed within a 3-month period on average, might have a synergistic and long-term effect on the VM, explaining the observed lack of stability after a year from the baseline.

We subsequently evaluated the association between reported symptoms and VM stability. Overall, higher stability of the VM was inversely associated with the persistence of dryness and abnormally high pH. Among the eight different symptoms reported, we showed that dryness is also associated with lower resistance in the VM, when comparing baseline with T1. Dryness is overrepresented among high diversity, lactobacilli deficient VMs at baseline, and such VMs tend to have the largest changes when evaluated 3 months later, both in women treated for gynecologic cancer and in healthy controls. Moreover, high pH was also correlated with low resistance, an indication that a low acidity, dysbiotic VM is prone to larger shifts in community states and lacking in equilibrium.

Our results highlight the importance of overall stability in the individual subject VM in staving off symptoms, rather than a specific community structure, with the exception of the typical lactobacilli dominant community types. In other words, asymptomatic women might express a variety of taxonomic distributions in their VMs, and stability over time tends to associate with the lack of persistent symptoms.

There are several limitations to this study that should be noted: (a) low statistical resolution due to the small sample size and heterogeneity of the population in terms of types of neoplasm and treatment modules (b) functional redundancy of the vaginal microbiome which overwhelm the taxonomic distributions, and yet unknown functional differences of subspecies or strains within the same species, not readily resolved with 16S rRNA gene amplicon sequences. Larger samples and metagenomic functional analyses are required to overcome these limitations in the future.

### Clinical Significance and Future Directions

There is a high prevalence of vaginal toxicities and symptoms associated with changes in the vaginal environment in both women treated for gynecologic malignancies and otherwise healthy postmenopausal women. However, the changes are more prevalent and persistent in women with gynecologic cancers. Larger longitudinal studies would help us better understand what constitutes a ‘healthy’ vaginal microbiota, as well as understanding the fluctuations that commonly occur in the postmenopausal state over time compared to changes in the vagina after cancer therapies. The community states and specific taxa found to be associated toxicities/symptoms by our study provide candidates and hypotheses to target for functional metagenomics in the future. These insights will lay the groundwork for novel and targeted interventional approaches, like probiotics, to alleviate the morbidity and mortality associated with vaginal dysbiosis in gynecologic cancer patients.

## Data Availability Statement

The datasets presented in this study can be found in online repositories. The names of the repository/repositories and accession number(s) can be found below: https://www.ncbi.nlm.nih.gov/, PRJNA448161.

## Ethics Statement

The studies involving human participants were reviewed and approved by Emory University Institutional Review Board. The patients/participants provided their written informed consent to participate in this study.

## Author Contributions

DB and KK conceived and designed the study, acquired resources and funding, and edited the original draft. RM, TE, JS, MD, JA, NK, PP, and IS consented and recruited patients. DT performed the formal analysis, data curation, DNA extraction, library preparation for sequencing and wrote the original draft. All authors contributed to the article and approved the submitted version.

## Funding

This study was partially supported by a Winship Cancer Institute grant (project number #00075389) to DB and U.S. National Science Foundation award no.1759831 to KK.

## Conflict of Interest

The authors declare that the research was conducted in the absence of any commercial or financial relationships that could be construed as a potential conflict of interest.

## Publisher’s Note

All claims expressed in this article are solely those of the authors and do not necessarily represent those of their affiliated organizations, or those of the publisher, the editors and the reviewers. Any product that may be evaluated in this article, or claim that may be made by its manufacturer, is not guaranteed or endorsed by the publisher.
