# Supplementary material for: Changes in the Vaginal Microbiome and Associated Toxicities Following Radiation Therapy for Gynecologic Cancers
Source: Front Cell Infect Microbiol. 2021 Oct 27;11:680038. doi: 10.3389/fcimb.2021.680038 (PMC8580013; doi:10.3389/fcimb.2021.680038)
Supplement: Supplementary file 2 [file DataSheet_2.docx]

**
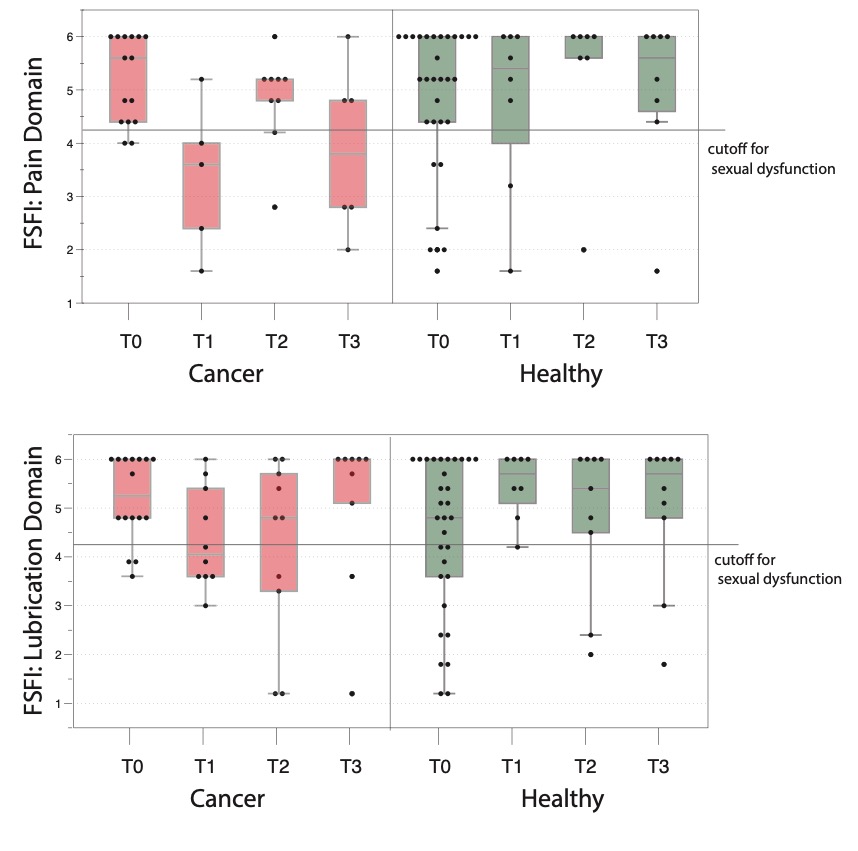
**

**Supplementary Figure 1:** Female sexual function domains scores for lack of lubrication and vaginal pain in sexually active subjects.


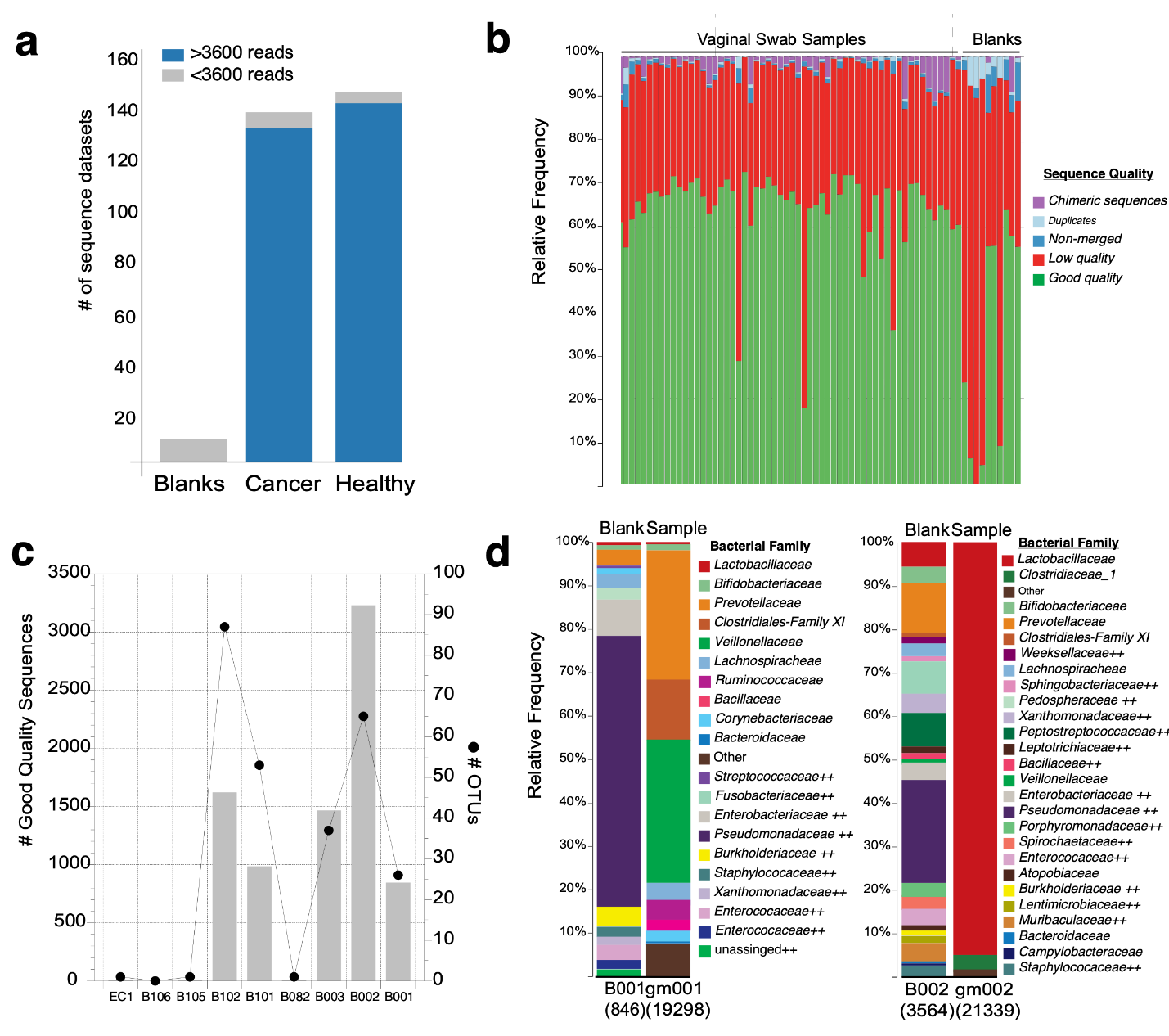


**Supplementary Figure 2:** Quality summary statistics for 16S rRNA gene amplicon datasets for 15 blank samples. (a) Number of datasets with >3600 reads, which was the yield quality cutoff used in this study (b) Distribution of chimeric, duplicated, low QC score and good quality reads among representative sequence datasets from vaginal swabs and blank samples. (c) Number of good quality sequences and bacterial OTUs (genera) obtained for each blank sample (d) Taxonomic distribution of blank samples in comparison to the vaginal swabs, which were collected in parallel.

A
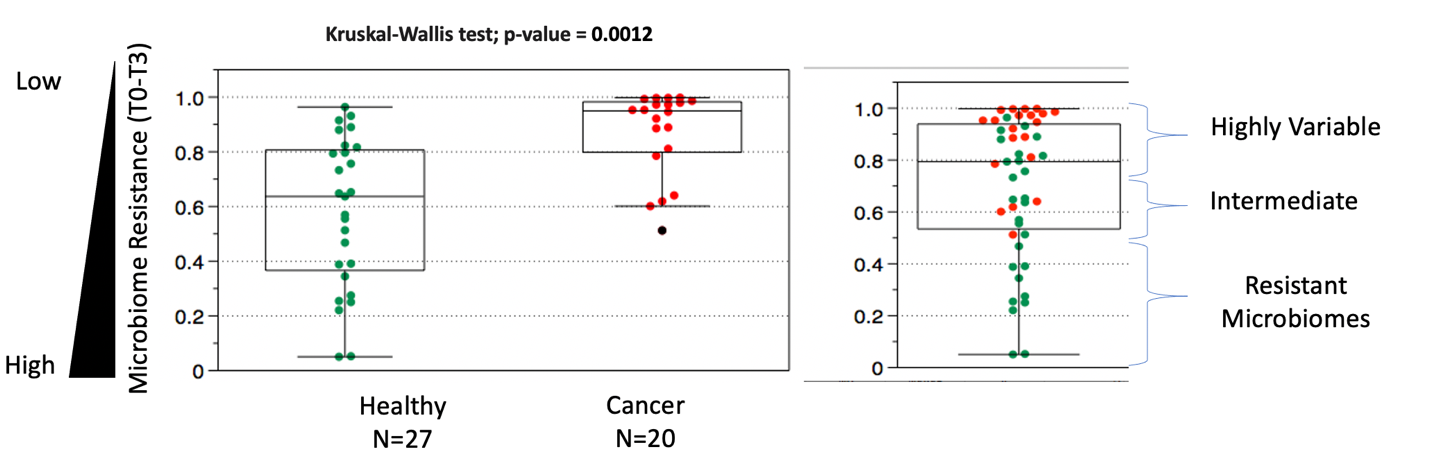


B


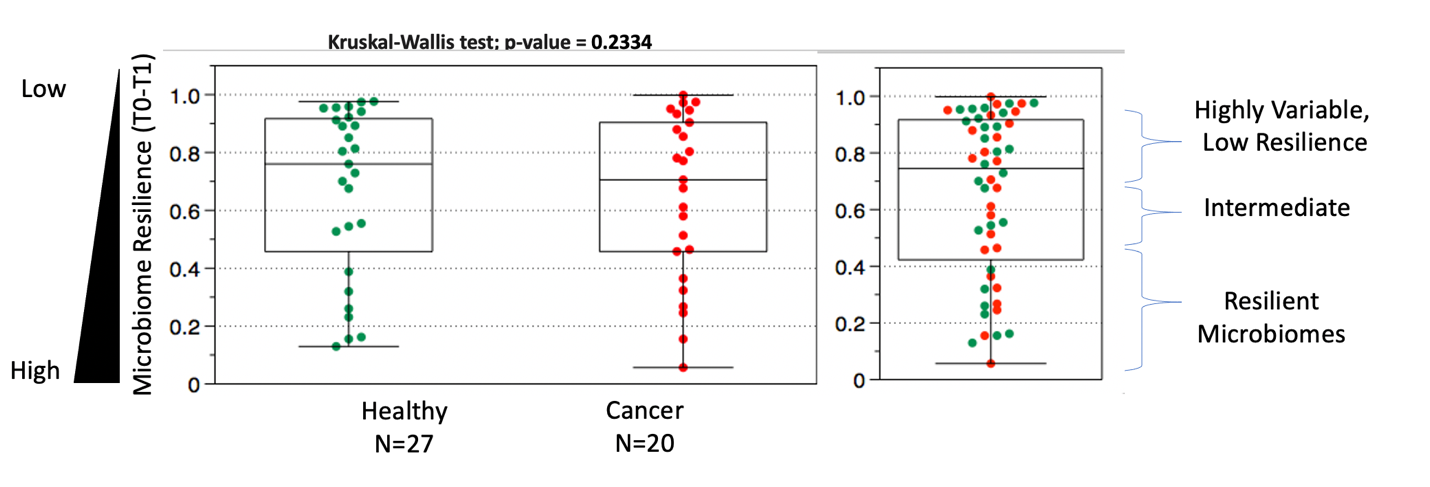


**Supplementary Figure 3.** Stability metrics of the vaginal microbiome in gynecologic cancer and healthy postmenopausal women. Overall stability (comparisons across 4 time points is shown in main figure 4. (A) Resistance of the vaginal microbial communities as estimated by the Bracy-Curtis distances between T0 and T1 for each subject. The resistance metric aims to quantify the magnitude of shifts observed from baseline to T1, right after the completion of radiation for cancer subjects. Healthy controls are assumed to have had no major therapeutic interventions. (B) Resilience of the vaginal microbiome communities, estimated from the Bray-Curtis distances between T0 and T3. The resilience metric aims to quantify the magnitude of the VM shifts from baseline (before the radiation therapy intervention for cancer patients) until one year later (when all interventions were completed for at least 9 months).
